# Interventions for waterpipe tobacco smoking prevention and cessation: a systematic review

**DOI:** 10.1038/srep25872

**Published:** 2016-05-11

**Authors:** Mohammed Jawad, Sena Jawad, Reem K. Waziry, Rami A. Ballout, Elie A. Akl

**Affiliations:** 1 Department of Primary Care and Public Health, Imperial College London, London, United Kingdom; 2 Academic Unit of Primary Care and Population Sciences, University of Southampton, Southampton, United Kingdom; 3 Nuffied Department of Primary Care Health Sciences, University of Oxford, Oxford, United Kingdom; 4 Faculty of Health Sciences, American University of Beirut, Beirut, Lebanon; 5 Faculty of Medicine, American University of Beirut, Beirut, Lebanon; 6 Department of Internal Medicine, American University of Beirut, Beirut, Lebanon; 7 Department of Clinical Epidemiology & Biostatistics, McMaster University, Hamilton, Canada

## Abstract

Waterpipe tobacco smoking is growing in popularity despite adverse health effects among users. We systematically reviewed the literature, searching MEDLINE, EMBASE and Web of Science, for interventions targeting prevention and cessation of waterpipe tobacco smoking. We assessed the evidence quality using the Cochrane (randomised studies), GRADE (non-randomised studies) and CASP (qualitative studies) frameworks. Data were synthesised narratively due to heterogeneity. We included four individual-level, five group-level, and six legislative interventions. Of five randomised controlled studies, two showed significantly higher quit rates in intervention groups (bupropion/behavioural support versus placebo in Pakistan; 6 month abstinence relative risk (RR): 2.3, 95% CI 1.4–3.8); group behavioural support versus no intervention in Egypt, 12 month abstinence RR 3.3, 95% CI 1.4–8.9). Non-randomised studies showed mixed results for cessation, behavioural, and knowledge outcomes. One high quality modelling study from Lebanon calculated that a 10% increase in waterpipe tobacco taxation would reduce waterpipe tobacco demand by 14.5% (price elasticity of demand −1.45). In conclusion, there is a lack of evidence of effectiveness for most waterpipe interventions. While few show promising results, higher quality interventions are needed. Meanwhile, tobacco policies should place waterpipe on par with cigarettes.

Waterpipe tobacco smoking is a growing public health concern. Recent national data from the Global Youth Tobacco Survey (aged 13–15 years) measured that past-30 day waterpipe tobacco use is highest in Lebanon (37%), the West Bank (33%) and Latvia (23%)^1^. Data from the National Youth Tobacco Survey (aged 11–18 years) in the US showed an increase in past-30 day waterpipe tobacco use from 4.1% in 2011 to 9.4% in 2014^2^. In Western countries, users are more likely to be young, non-white males of high socioeconomic status^3^. Given these data, waterpipe tobacco smoking is likely to undermine the World Health Organisation’s Global Action Plan one of the aims of which is to achieve a 30% worldwide relative reduction in tobacco use by the year 2025^4^.

Worrisome reports of increasing prevalence are compounded by the fact that waterpipe tobacco smoking exposes users to significant levels of toxicants. Waterpipe tobacco contains significant quantities of tar, nicotine, heavy metals and tobacco-specific nitrosamines^5^,^6^,^7^. Waterpipe tobacco is mixed with sugars and flavours, which additionally produce volatile aldehydes when heated^8^. Furthermore the product is heated by charcoal which is a source of carbon monoxide, polycyclic aromatic hydrocarbons and other carcinogens^9^,^10^. Expectedly, systematic reviews of the health effects of waterpipe tobacco smoking show significant associations with lung cancer, respiratory diseases, low birth weight and periodontal diseases^11^,^12^.

A recent review on the motives, attitudes and beliefs towards waterpipe tobacco smoking identified that waterpipe users generally perceive it to be relatively harmless compared with cigarette smoking^13^. Underlying reasons for this include widespread social acceptance, relaxation and pleasure, and confidence in the ability to quit.

The Eastern Mediterranean Region, where waterpipe tobacco smoking prevalence is highest, is projected to be the only region in the world where no countries are expected to reach the WHO Global Action targets for tobacco reduction among males under existing tobacco control policies^14^. In view of these findings, effective clinical, public health, and health systems interventions are needed to prevent initiation and promote cessation of waterpipe tobacco smoking. A Cochrane Review published in 2015 found only three randomised controlled trials for waterpipe cessation^15^. The objective of this study was to systematically review the medical literature for interventions targeting the prevention and cessation of waterpipe tobacco smoking. This review builds upon the Cochrane review by additionally including prevention interventions, legislative interventions, and by considering a broader range of study designs.

## Methods

Our protocol is registered on PROSPERO (CRD42014010296) and can be found at http://www.crd.york.ac.uk/PROSPERO/display_record.asp? ID=CRD42014010296. The main change to the protocol was the inclusion of qualitative studies. This was due to the low number of initially included studies in this review.

### Eligibility criteria

#### Our inclusion criteria included:

- Study design: randomised controlled trials, non-randomised trials (e.g., pre-post studies), observational studies (e.g., interrupted time series, cohort studies), and qualitative studies published in English or Arabic. Studies did not need a minimum sample size to be included.
- Population: individuals, groups, or communities at risk of, or practicing waterpipe tobacco smoking without geographical restrictions.
- Intervention: clinical, public health, or health service interventions for the prevention or cessation of waterpipe tobacco smoking.
- Control: another intervention or no intervention (for randomised and non-randomised trials)
- Outcomes of interest: any validated or non-validated instrument which captured the knowledge, attitudes, health behaviour (e.g. avoidance, reduction, cessation), and health effects related to waterpipe tobacco smoking. Additional outcomes included adverse effects specific to the intervention or control, and substitution to different tobacco products.

We excluded case-control studies, ecological studies, case reports, case series, experimental lab studies, studies not reporting original research (e.g., commentaries, reviews), and legal proceedings. We excluded studies that did not distinguish waterpipe tobacco smoking from other forms of tobacco use, unless study authors provided us with additional waterpipe-tobacco specific data. We excluded studies about forms of tobacco smoking other than waterpipe even if conducted among waterpipe users. We also excluded studies reported as abstracts and for which we could not identify a full text after consultation of a medical librarian or contact with the corresponding author.

#### Search strategy

In May 2015 we searched the following electronic databases with no language or date restrictions: MEDLINE (access via OVID), EMBASE (access via OVID) and the ISI Web of Science. We used synonyms and spelling variations of the terms “waterpipe,” “hookah,” “shisha,” “narghile” and other culturally-specific terms (Supplementary Table S1). Two medical librarians reviewed and commented on the search strategy.

In addition, we hand-searched the citation lists of included studies and of review articles and used the Google Scholar function to find articles that cited relevant studies. We requested additional unpublished data from corresponding authors of studies which failed to distinguish between waterpipe tobacco smoking and other forms of tobacco use. We also enquired about the availability of data from corresponding authors of studies on waterpipe tobacco prevention or cessation intervention protocols, and contacted corresponding authors for studies which we could not find a full text. We also put in place a literature surveillance process in order to find additional studies throughout the development of this review.

#### Selection process

Two reviewers (MJ and RAB) screened in duplicate and independently the title and abstract of captured citations to identify potentially eligible studies. We retrieved full texts of studies considered potentially eligible by at least one reviewer. Two reviewers (MJ and SJ) then screened in duplicate and independently the full texts using a standardised and pilot-tested screening form. They resolved disagreements by discussion, and when needed with the help of a third reviewer (EAA).

#### Data abstraction

Two reviewers (MJ and SJ) abstracted in duplicate and independently data from each eligible study using a standardised and pilot-tested data abstraction form. They resolved disagreements by discussion, and when needed with the help of a third reviewer (EAA). We abstracted information on study design, participants (including selection criteria), the intervention, the control, outcomes, sources of funding, and declarations of interest.

#### Risk of bias assessment

For randomised trials, we used the Cochrane risk of bias framework, as outlined in the Cochrane Handbook for Systematic Reviews of Interventions^16^. The framework includes six domains: random sequence generation, allocation concealment, blinding, completeness of data, selective outcome reporting and other bias. For non-randomized studies, we used a framework suggested by the Grading of Recommendations Assessment, Development and Evaluation (GRADE) working group^17^. The framework includes five domains: developing and applying appropriate eligibility criteria, measurement of exposure, measurement of outcome, controlling for confounding, and completeness of data. For qualitative studies, we used the Critical Appraisal Skills Program (CASP) framework^18^. The framework’s ten items include the clarity of aims and objectives, the appropriateness of qualitative research, the appropriateness of the research design, the appropriateness of the recruitment strategy, the appropriateness of data collection, consideration of the relationship between the researcher and the participants, consideration of ethical issues, sufficient rigour in data analysis, a clear statement of findings, and a discussion about the value of research.

#### Data analysis

We calculated the kappa statistic to evaluate the agreement between the two reviewers assessing full texts for eligibility. We grouped studies and analysed them by one of three intervention types: individual-level (clinical) interventions, group-level (public health) interventions, and legislative (health systems) interventions. When more than one study evaluated the same intervention type, we considered conducting meta-analyses for the outcomes of interest using a random effects model to account for the heterogeneity between studies. For studies reporting more than one measure for the cessation outcome measure, we used the primary measure most consistently used across studies. We undertook a narrative synthesis for data we could not quantitatively synthesise. We conducted all statistical analyses using Stata 12 (StataCorp). We then rated the overall quality of evidence for each intervention using the GRADE approach^17^,^19^,^20^.

## Results

### Description of included studies

**Figure 1:**
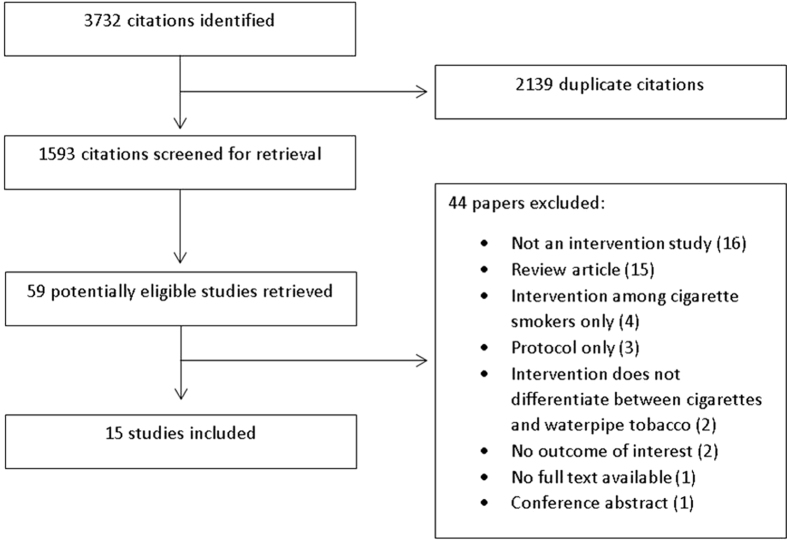
Study flow diagram which shows the process of screening and selecting studies for inclusion in this systematic review.

Figure 1 presents the study flow. Of 59 potentially eligible studies, we excluded 44 for the following reasons: not an interventional study (16), review article (15), intervention among cigarette smokers only (4), protocol (3), intervention does not differentiate between cigarettes and waterpipe tobacco (2), no outcome of interest (2), no full text available (1) and conference abstract (1). The kappa statistic between the two reviewers assessing full texts for eligibility was 0.86.

Supplementary Table S2 presents the detailed characteristics of the 15 included studies. Four were individual-level (clinical) behavioural interventions^21^,^22^,^23^,^24^, five were group-level (public health) behavioural interventions^25^,^26^,^27^,^28^,^29^, and six were legislative (health systems) interventions^30^,^31^,^32^,^33^,^34^,^35^. Five studies were randomised controlled trials^21^,^22^,^23^,^25^,^28^, five were pre-post studies^24^,^26^,^27^,^29^,^30^, four were qualitative studies^31^,^32^,^33^,^34^ and one was an economic modelling study^35^. Four studies were conducted in England^31^,^32^,^33^,^34^, two were conducted in Pakistan^22^,^27^, and the remainder were conducted in Egypt^25^, Germany^26^, India^30^, Israel^24^, Lebanon^35^, both Lebanon and Qatar^28^, Saudi Arabia^29^, Syria^21^ and the US^23^. Two studies tested the effect of cigarette-specific interventions on waterpipe tobacco smoking^22^,^26^. Supplementary Table S3 details the risk of bias assessment for each study, while Table 1 provides a summary of that assessment.

### Synthesis of results

Supplementary Table S4 details individual results of each study. We divided our results in three subcategories based on intervention type: individual-level (clinical) behavioural interventions, group-level (public health) behavioural interventions or legislative (health systems) interventions. We considered meta-analyses for individual-level (clinical) behavioural interventions however they were not comparable in terms of population, intervention and outcome measures. As such, a narrative synthesis was conducted.

#### Individual-level interventions

Four studies evaluated individual-level waterpipe interventions. The first study conducted by Asfar *et al*. in Syria between 2007 and 2008 was a pilot, two-arm, parallel group, randomised, open label trial among 50 waterpipe-only smokers^21^. It compared an intensive behavioural intervention (intervention group) with a brief behavioural intervention (control group). The relative risk of self-reported, carbon monoxide (CO) verified (<10ppm) prolonged abstinence at three months post-cessation in the intervention group was 1.46 (95% CI 0.69–3.09). For secondary cessation outcomes, the relative risk for seven day point prevalence was 1.34 (95% CI 0.57–3.35) and for continuous abstinence was 1.07 (95% CI 0.27–4.42).

The second study conducted by Dogar *et al*. in Pakistan between 2010 and 2011 was a three-arm, cluster, randomised non-inferiority trial among 1181 cigarette-only smokers, 200 waterpipe-only smokers and 460 mixed smokers^22^. The three arms were a brief behavioural intervention (BSS), a brief behavioural intervention plus bupropion for seven weeks (BSS+), and standard care (control group). Compared to the control group, the relative risk of self-reported, CO verified (<10ppm) smoking abstinence at 25 weeks by waterpipe-only smokers was 2.2 (95% CI 1.3–3.8) in the BSS group and 2.5 (95% CI 1.3–4.7) in the BSS+ group. Combining the BSS and BSS+ group versus placebo, the relative risk was 2.3 (95% CI 1.4–3.8).

The third study conducted by Lipkus *et al*. in the US between 2009 and 2010 was a randomised controlled online intervention among 91 college/university students who currently smoked waterpipe in central North Carolina^23^. Students were randomised to non-health-related and health-related information about waterpipe (intervention group) or to non-health-related information about waterpipe only (control group). The relative risk of self-reported waterpipe cessation at six months was 1.46 (95% CI 0.81–2.62) in the intervention group. This was not biochemically verified.

The fourth study conducted by Essa-Hadad *et al*. in Israel between 2007 and 2010 was an uncontrolled pre-post online intervention among 225 Arab adults across several universities^24^. Students received tailored text- and video-based health education material depending on their waterpipe smoking status. The prevalence of past-seven day waterpipe use decreased from 58.2% at baseline to 22.2% at one month follow up (p = 0.001).

#### Group-level interventions

Five studies evaluated group-level waterpipe cessation interventions. The first study conducted by Mohlman *et al*. in Egypt between 2004 and 2005 was a cluster randomised controlled intervention among 7657 residents in villages across the Qalyubia governorate^25^. Villages were randomised to receive a behavioural intervention through a variety of activities engaging school students, places of worship, and adult women (intervention group), or no intervention at all (control group). The relative risk of waterpipe cessation among current male users at one year was 3.25 (95% CI 1.39–8.89) in the intervention group. This was not biochemically verified. The intervention group saw a 25.6% relative increase (38.3%–48.1%) in the belief that waterpipe smoking is not less harmful than cigarettes compared to a 10.8% relative increase (40.6%–44.9%) in the control group. Furthermore, the intervention group experienced a 20.9% relative decrease (48.8%–38.6%) in not knowing whether waterpipe smoking was less harmful compared to an 11.2% relative decrease (48.5%–43.1%) in the control group.

The second study conducted by Nakkash *et al*. in Lebanon and Qatar between 2011 and 2012 was a national cluster randomised controlled intervention among 1857 students in grades 6, 7 or 8^28^. Schools were randomised to receive ten sessions over ten weeks covering knowledge on waterpipe (4 sessions), refusal skills and social promise (3 sessions), decision making (2 sessions) and media literacy (one session). Results were only available for the Lebanon schools, where post-intervention outcomes were assessed a few days after the intervention. Compared to the control group, the relative risk of past-30 day waterpipe use was 0.99 (95% CI 0.82–1.20), and of waterpipe cessation among past-30 day users at baseline was 1.50 (95% CI 0.89–2.53), in the intervention group. These were not biochemically verified. Furthermore, compared to the control group the relative risk of having increased waterpipe knowledge was 1.51 (95% 1.37–1.66) and of having a “healthy” waterpipe attitude was 1.29 (95% CI 1.13–1.47) in the intervention group.

The third study conducted by Stamm-Balderjahn *et al*. in Germany between 2007 and 2008 was a controlled, non-randomised study among 760 school students aged 12–22 years^26^. Students were non-randomly assigned to receive health information relating to tobacco smoking (discussions with a physician and a patient suffering from a tobacco-related illness, as well as measuring their own lung function) or no intervention at all. The main waterpipe-specific outcome was prevention from waterpipe tobacco smoking initiation among non-waterpipe smokers, which was measured six months after the intervention and not biochemically verified. Compared to the control group, the odds of staying abstinent among non-waterpipe smokers was 3.64 (95% CI 1.32–10.03) in the intervention group.

The fourth study conducted by Anjum *et al*. in Pakistan in 2006 was an uncontrolled pre-post study among 646 school students aged 14–19 years^27^. Students received eight interactive sessions on waterpipe tobacco smoking. Outcomes were measured two months after the intervention. Comparing pre- and post-intervention surveys, there was no change in prevalence of waterpipe smoking (ever smoker 27% vs. 24%, p = 0.37; current smoker 17% vs. 14%, p = 0.27). There was a significant decrease in waterpipe smokers who shared the waterpipe with others (76% vs. 68%) and smoked waterpipe with friends or family (91% vs. 85%) (both p < 0.05). There was no change in frequency or location of waterpipe use between the two surveys. Of the multitude of outcomes relating to knowledge, attitudes and beliefs, (detailed in Supplementary Table S4), there was a significant increase in those who wanted to quit waterpipe (32% pre-test vs. 53% post-test), in those who believed waterpipe is addictive (54% vs. 68%), more addictive than cigarettes (11% vs. 32%), more harmful than cigarettes (16% vs. 45%), more socially acceptable than cigarettes (58% vs. 80%), that waterpipe is Pakistani cultural heritage (29% vs. 58%), and that girls are more comfortable smoking waterpipe than cigarettes (66% vs. 79%) (all p < 0.05). There was also a significant increase in those who believed waterpipe was associated with oral infections (12% vs. 17%), bladder cancer (19% vs. 33%), lip cancer (35% vs. 61%) and infertility (10% vs. 38%) (all p < 0.05). However there was significant decrease in those who believed waterpipe was associated with cardiovascular disease (24% vs. 10%) and high blood pressure (31% vs. 16%) (both p < 0.05).

The fifth study conducted by Quadri *et al*. in Saudi Arabia in 2013 was an uncontrolled pre-post study among 1051 school/university students aged 15–25 years^29^. Students received one lecture, an educational brochure and a question-answer session about the risk factors for oral cancer. A knowledge score that waterpipe smoking was a risk factor for oral cancer increased from 0.80 (SD 0.34) to 0.98 (SD 0.13), assessed immediately after the intervention.

#### Legislative interventions

Six studies assessed legislative interventions. The first four studies qualitatively looked at the impact of the smokefree law (public indoor smoking ban) in the United Kingdom, which was implemented on 1^st^ July 2007^31^,^32^,^33^,^34^. In one study conducted by Highet *et al*. among Bangladeshi smokers in the United Kingdom interviewed pre- and post-implementation of the smokefree law, one participant explained how the smokefree law ceased his cigarette use but increased his waterpipe tobacco consumption instead: “I also use shisha as a substitute for coming off cigarettes, some people use nicotine patches and all that, I find shisha more effective”^31^. This was the only reference to waterpipe and most data related to changes in cigarette use.

A second study conducted by Lock *et al*. among ethnic minorities in north London, again interviewed pre- and post-implementation of the smokefree law, highlighted the behaviour change of female, Somali waterpipe smokers^32^. Prior to the smokefree law, Somali females were able to privately smoke inside waterpipe-serving premises; a preference for them as smoking among females is considered a community taboo. The smokefree law prohibited indoor use of these premises, resulting in Somali females taking measures to conceal themselves, use illicitly-run waterpipe-serving premises to smoke indoors, or smoke at home instead. One young Somali female said: “For girls, they cannot sit outside [waterpipe-serving premises to smoke]. They feel a bit embarrassed. A friend - a family friend, like, someone might see them and tell the family. So what happened was, they put hoods on, a bit of clothing on. Now they face on the wall, and they’re just smoking, but they cannot sit there for a long time. And when they’re, like, smoking the Shisha, they’re not feeling comfortable.”

The findings from Lock *et al*. are supported by the third study conducted by Jawad *et al*. among ethnic minorities in London, where some regular waterpipe users adapted to the smokefree law by smoking at home instead of at cafes, resulting in increased waterpipe consumption^33^.

A fourth study involved interviewing local government staff members involved in enforcing laws on waterpipe-serving premises in London six years after implementation of the smokefree law^34^. Staff working in highly dense areas of waterpipe-serving premises described how the smokefree law resulted in considerable extra work against waterpipe-serving premises due to poor compliance. It was believed that fines for breaching the smokefree law (ranging from £300 to £1500 per premise) were not a strong enough deterrent to promote compliance with the smokefree law.

A fifth study conducted by Deshpande *et al*. in India between 2008 and 2009 was a pre-post study in 25 hospitality venues in Mumbai, of which five were waterpipe-serving premises^30^. All hospitality venues were subject to the implementation of the smokefree law on 2^nd^ October 2008. The main outcomes were PM_2.5_ levels and active smoker density (calculated by dividing the number of people smoking per room by the room volume). PM_2.5_ levels decreased in all hospitality venues except in waterpipe-serving premises, which increased by a mean 973 ug/m^3^ pre-ban to 1267 ug/m^3^ post-ban (a 30% increase). Active smoker density decreased to zero in all hospitality venues except waterpipe-serving premises, where it increased to 3.08 burning cigarettes per 100 cubic meters volume.

The sixth study conducted by Salti *et al*. in 2005 among adults in Lebanon used cross-sectional data from the Household Living Conditions Survey. This national survey contains price indices for different goods and services to model the effect of increased waterpipe tobacco taxation on home-based waterpipe tobacco consumption^35^. The own-price elasticity of demand for waterpipe tobacco was −1.45 (SD 0.007), meaning that a 10% rise in the price of waterpipe tobacco would result in a 14.5% relative decrease in its consumption.

## Discussion

This systematic review reports on interventions for waterpipe tobacco smoking prevention and cessation. Of seven intervention studies that reported on cessation outcomes, all participants in intervention groups had higher cessation rates than control groups, although this was only significant in three studies^22^,^24^,^25^. We go beyond the Cochrane review on interventions for waterpipe cessation^15^ by providing insight on how behavioural interventions can prevent waterpipe initiation and change knowledge about waterpipe, and how policy measures affect waterpipe-serving premises and consumer behaviour. Few studies show promising results in favour of waterpipe tobacco interventions, although the pilot nature and high risk of bias of most studies suggests that better designed and larger trials are needed for firmer conclusions to be made.

The degree to which waterpipe-specific interventions are needed is uncertain, considering two studies with significant results did not use waterpipe-specific interventions and instead delivered cigarette interventions to waterpipe smokers^22^,^26^. Non-specific interventions may work for waterpipe smokers given they are likely to address the shared determinants of waterpipe and cigarette smokers, such as nicotine dependence, harm to self, and harm to others. However, waterpipe-specific interventions may work better as they can further include unique socio-cultural dimensions of use such as the emphasis on tobacco flavours, long preparation and smoking times, and sharing with peers^33^,^36^.

Except for one study that delivered an intervention involving behavioural support with bupropion^22^, data are lacking on the effectiveness of pharmacotherapies for waterpipe tobacco smoking cessation. Cessation services faced with waterpipe tobacco smokers should be prudent to the fact that waterpipe and cigarette interventions may interfere with (or even undermine) one another^37^. For example, a cigarette cessation trial among 50 users in Syria resulted in three participants in the intervention group initiating waterpipe tobacco use for the first time during their cigarette quit attempt^38^. Worryingly, two longitudinal studies among young people in Denmark and Jordan have shown that waterpipe tobacco use may be a risk factor future cigarette initiation^39^, or increased cigarette frequency^40^, respectively.

In addition to the need to develop well-designed behavioural interventions, there have been widespread calls by tobacco control researchers to address waterpipe tobacco smoking in a stronger policy framework. This review gives insight into two of the most effective tobacco control interventions for cigarettes: taxation and smokefree laws. According to our included study on waterpipe tobacco taxation^35^, a 10% increase in waterpipe tobacco taxation should reduce demand of waterpipe tobacco by 14%, but may also increase the consumption of imported cigarettes by 1.5%. It is not surprising that waterpipe tobacco and cigarettes act as substitute goods, although any waterpipe policy measures should consider the wider impact on other tobacco products.

This review anecdotally identifies difficulties in implementation of smokefree legislation among waterpipe-serving premises in India and England, further insight into which is provided by analyses of qualitative studies. This is in spite of English and Indian laws containing no smokefree exemptions for waterpipe-serving premises^41^. A recent review of tobacco control laws from 62 countries worldwide suggested that a significant minority of countries may have exemptions for health warnings and misleading descriptors on waterpipe tobacco^41^. Moreover, several large cities in the US are known to have smokefree law exemptions for waterpipe-serving premises^42^, despite reports of carbon monoxide poisoning^43^ and poor air quality in these venues^44^,^45^,^46^. Europe is set to ban flavoured tobacco under the new EU Tobacco Control Directive, but this ban will exempt waterpipe tobacco and be specific only to cigarettes and hand-rolled tobacco^47^. Data from the UK suggest some waterpipe-serving premises deliberately and recurrently breach tobacco control legislation due to high profit margins^34^. In light of our findings there is a need for heightened legislative attention towards waterpipe tobacco smoking.

This review benefits from its systematic approach in line with recommendations of the Cochrane Handbook for Systematic Reviews of Interventions^16^. To our knowledge we are the first to review interventional studies for waterpipe tobacco smoking beyond randomised controlled trials. The inclusion of non-randomised studies strengthens the review by virtue of enabling a holistic overview of the literature. In particular, our included qualitative studies may serve to generate novel hypotheses about the effectiveness of existing tobacco control laws on emerging tobacco products such as waterpipe tobacco. In terms of weaknesses, we only searched for studies in the English and Arabic language and may have omitted studies from other languages. Given the fact that most of the included studies were small in design, we cannot exclude small study effects and publication bias. Many non-randomised studies failed to use control groups and failed to control for confounding, which has introduced an overall high risk of bias. Another weakness includes the inability to meta-analyse results due to heterogeneity of interventions.

Our review has several implications. Clinically, our findings show a lack of evidence of high enough quality to recommend behavioural and pharmacological interventions for patients seeking waterpipe cessation. However, these interventions could be considered given there is no evidence they cause harm. It is also uncertain how health practitioners aiming to reduce the prevalence of waterpipe smoking in their communities can proceed with interventions. Legislatively, we recommend that waterpipe tobacco should be enforced on equal footing as cigarettes, especially with regards to implementation of smokefree laws and raising of taxes. Waterpipe-serving premises should be under routine surveillance by local government as it appears they are known for undermining tobacco control policies. Our findings also have research implications. There is a need for better quality waterpipe interventions, including the need to explore the effectiveness of different pharmacotherapies and other forms of behavioural interventions such as group sessions. The fact that outcomes were extremely heterogeneous across studies calls for their standardisation and/or validation in future studies. For example, waterpipe cessation interventions used immediate^28^, three month^21^, six month^22^,^23^ and 12 month^25^ follow up measures as a primary outcome, making comparisons challenging. Future cessation interventions should be compared to unassisted quitting rates, in order to inform design of tailored waterpipe cessation services. Finally, legislative interventions should be evaluated in order to inform health policy makers.

To conclude, there is a lack of evidence of effectiveness for most waterpipe interventions although few behavioural interventions show promising results in different settings. Further high quality interventions are needed, including pharmacotherapy cessation interventions, to promote waterpipe tobacco cessation. Legislative interventions anecdotally appear ineffective and this should be addressed by evaluating current waterpipe tobacco legislation and placing it on par with cigarettes.

## Additional Information

**How to cite this article**: Jawad, M. *et al*. Interventions for waterpipe tobacco smoking prevention and cessation: a systematic review. *Sci. Rep.* **6**, 25872; doi: 10.1038/srep25872 (2016).

## Supplementary Material

Supplementary Information

## Figures and Tables

**Table 1:**
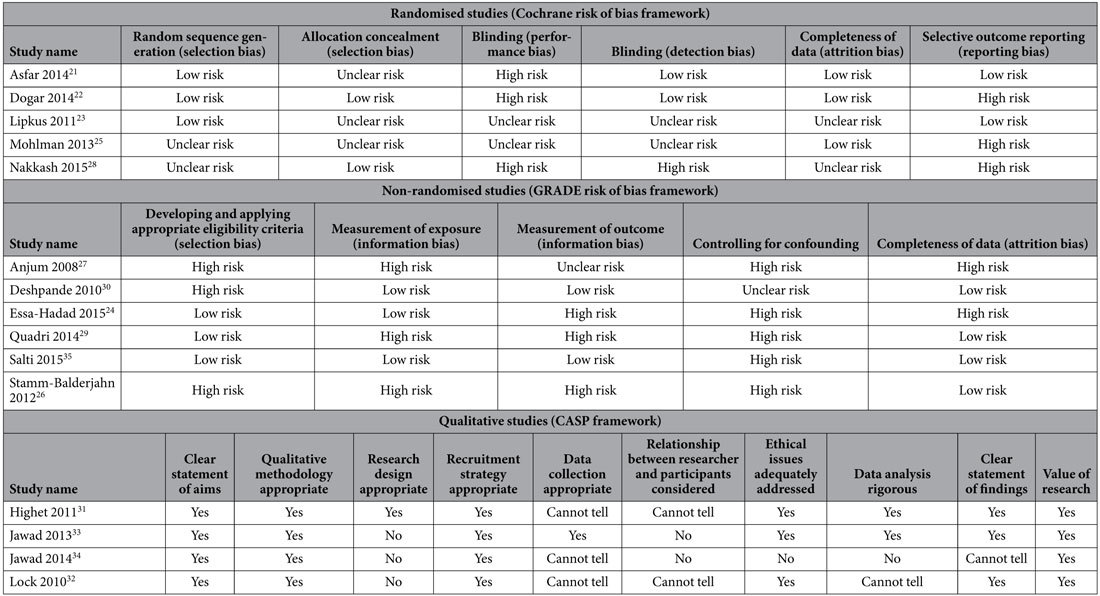
Summarised risk of bias for included studies (full data found in Supplementary Table S3).
