# Supplementary material for: Interventions for waterpipe tobacco smoking prevention and cessation: a systematic review
Source: Sci Rep. 2016 May 11;6:25872. doi: 10.1038/srep25872 (PMC4863147; doi:10.1038/srep25872)
Supplement: Supplementary Information [file srep25872-s1.pdf]

Interventions for waterpipe tobacco smoking prevention and cessation: a systematic review

Mohammed Jawad, Sena Jawad, Reem K Waziry, Rami A Ballout, Elie A Akl

Supplementary Table S1: Electronic search strategies

Database	Search strategy
**MEDLINE** (1950 onward)	Waterpipe*.mp.<br><br>“water pipe*”.mp.<br><br>shisha*.mp.<br><br>sheesha*.mp.<br><br>hooka*.mp.<br><br>huqqa*.mp.<br><br>guza*.mp.<br><br>goza*.mp.<br><br>narghil*.mp.<br><br>nargil*.mp.<br><br>arghil*.mp<br><br>argil*.mp<br><br>(hubbl* adj3 bubbl*).mp.<br><br>or/1-13
**EMBASE** (1988 onward)	Waterpipe*.mp.<br><br>“water pipe*”.mp.<br><br>shisha*.mp.<br><br>sheesha*.mp.<br><br>hooka*.mp.<br><br>huqqa*.mp.<br><br>guza*.mp.<br><br>goza*.mp.<br><br>narghil*.mp.

	nargil*.mp.<br><br>arghil*.mp<br><br>argil*.mp<br><br>(hubbl* adj3 bubbl*).mp.<br><br>or/1-13
**ISI the Web of Science**	(waterpipe* OR "water pipe*" OR shisha* OR sheesha* OR hooka* OR huqqa* OR guza* OR goza* OR narghil* OR nargil* OR argil* OR arghil* OR (hubbl* SAME bubbl*)) AND (smoking OR smoke OR health OR disease OR cancer* OR malignan* OR lung* OR pulmonary OR heart OR cardiac OR vascular OR stroke) (in Title or Topic

Interventions for waterpipe tobacco smoking prevention and cessation: a systematic review

Mohammed Jawad, Sena Jawad, Reem K Waziry, Rami A Ballout, Elie A Akl

Supplementary Table S2: Characteristics of included studies

Table 1: Characteristics of randomized studies

Study Name, Funding and Conflicts of Interest	Study Design	Participants	Intervention	Control	Outcomes assessed
Asfar 2014<br><br>Funding: Supported by a start-up grant (SUG) for tobacco related research from the Initiative for Cardiovascular Health Research in the Developing Countries (IC-Health). Also partially supported by the National Institute on Drug Abuse (NIDA) grants R01 DA024876 and R01 DA035160.<br><br>No conflicts of interest declared.	Design: Pilot two-arm, parallel group, randomized, open label trial<br><br>Year of study: 2007-2008	Eligibility criteria:<br>- Adults >= 18 years old<br>- Smoked WP >= 3/week in the last year<br>- Non-cigarette smokers<br>- Interested in quitting<br>- Exclusion criteria: unable to understand the study consent and procedures<br><br>Characteristics:<br>- N=50, 94% male, mean age 30, 60% completed high school, 44% married, 60% employed, 90% Muslim<br><br>Setting: private hospital, smoking cessation clinic in<br><br>Country: Syria<br>City/region: Aleppo	Three 45-min, individual, in person educational/counselling sessions by trained physician plus five brief (10 min) phone calls: 1 day before quit date and day 1, 10. 21, 30 after quit day	Single 45-min, individual, in person educational /counseling session by trained physician plus three brief (10 min) phone calls: 1 day before quit date, and day 1 and 7 after quit day	Waterpipe cessation<br><br>**Primary efficacy outcome**: self-reported and CO verified (<10ppm) prolonged abstinence at 3 months post-cessation (complete abstinence after 2 week grace period following quit day)<br><br>**Secondary outcomes**: i) self-reported and CO verified (<10ppm) 7 day point prevalent abstinence (no WP use 7 days before F/U interview) ii) self-reported and CO verified (<=10ppm) continuous abstinence (no WP use since quit day)
Dogar 2014<br><br>Funded by the International Development Research Centre, Canada.<br><br>No conflicts of interest declared.	Design: Three arm cluster randomized controlled non-inferiority trial<br><br>Year of study: 2011-2012	Eligibility criteria:<br>- Adults aged over 18 years<br>- Suspected tuberculosis (cough >=3 weeks of unknown cause)<br>- Regular smokers (>= 1 cigarette or waterpipe per day)<br>- Excluded those requiring hospitalization or urgent medical attention	1) Two brief behavioural support cessations (first visit 30min, second on quit day 10min) (BSS)<br><br>2) Two brief behavioural support cessations (as above) plus bupropion for seven weeks (75mg/d for first week, 150mg/d for next six weeks) (BSS+)	Usual care and a self-help leaflet	Waterpipe cessation<br><br>CO verified (<10ppm) continuous smoking abstinence at 5 and 25 weeks post-intervention F/U visits

		Characteristics:<br>- N=1955, mean age (SD) 52 (14), 21% female, median household income (IQR) 81.4 (69.8) USD<br><br>Setting: 33 primary and secondary health centres<br><br>Country: Pakistan<br>City/region: Jhang and Sargodha districts			
Lipkus 2011<br><br>Funding: National Cancer Institute Grant (R01 CA114389); National Institute on Drug Abuse Grant (P30 DA023026).<br><br>No conflicts of interest declared.	Design: Randomised controlled behavioural intervention<br><br>Year of study: 2009-2010	Eligibility criteria:<br><br>- Enrolled in a 4-year college or university<br><br>- Aged 18 years or older<br><br>- Smoked waterpipe at least once during the last month<br><br>Characteristics<br>- N=91,mean age (SD): 20 (2) years, 24% female, 77% Caucasian, 34.4% senior year<br><br>Setting: six college and university campuses<br><br>Country: USA<br>Region: North Carolina	Participants in the experimental group were shown 20 MS PowerPoint slides that covered 1) what is a waterpipe and how it works (e.g., names it goes by, schematic of a waterpipe), 2) what is in waterpipe, focusing on the flavorings added to the tobacco, 3) who smokes waterpipe, concentrating on origins, spread, and use by subgroups, 4) amount of smoke inhaled by waterpipe in laboratory studies and in relation to smoking cigarettes, 5) production of tar, CO, and nicotine in waterpipe tobacco compared to cigarettes, 6) exposure levels of toxic compounds (e.g., aldehyde), and 7) health effects associated with waterpipe tobacco smoking (e.g., cancer, heart disease, infections). The average length of time reviewing these materials online was 7.5 min for the experimental condition.	Control participants reviewed eight slides that covered points one through three listed above only. After review of the educational materials, participants in both conditions completed the main study measures.<br><br>The average length of time reviewing these	<u>Waterpipe cessation</u><br><br>Not smoking waterpipe in last 30 days among those who smoked monthly at baseline

				materials online was 3.6 min for the control condition.	
Mohlman 2013<br><br>Funding: The Fogarty International Center of the U.S. National Institutes of Health (R01TW059444).<br><br>No conflicts of interest declared.	Design: Cluster randomized controlled behavioral intervention<br><br>Year of study: 2004-2006	Eligibility criteria:<br>- All household members aged over 12 years old, although results pertain only to adult males (females n for self-reported smoking too small)<br><br>Characteristics<br>- N=7657<br>-Intervention group: mean age 37 years, 45% male, 72% married, 42% illiterate, 87% employed<br>- Control group: mean age 36yrs, 45% male, 74% married, 41% illiterate, 88% employed<br><br>- No difference between groups, except slightly more married participants in controlled group<br><br>Setting: Villages Egypt that had between 10,000-20,000 inhabitants, at least one primary, preparatory, and secondary school, a public health clinic, a youth club, a mosque<br><br>Country: Egypt<br>Region: Qalyubia governorate	<u>Health promotion over a 12 month period simultaneously in all six villages</u><br>1) Primary school students participated in traditional and nontraditional activities aimed at preventing the initiation of smoking by deglamorizing tobacco use and teaching about its health hazards.<br><br>2) Preparatory and secondary school students engaged in an experiential learning program to develop social skills among teenagers to handle peer pressure to smoke.<br><br>3) Engaging mosques and churches in educating their communities about the hazards of smoking and ETS and in raising the issue of smoking as a sinful behaviour<br><br>4) Female social change agents (*raedat refeyat*) provided information to adult women in the home on the negative health effects of tobacco use and ETS.<br><br>5) They also taught these women how to better protect themselves and their children from ETS through a standardized message sensitive to cultural family dynamics.	No health promotion.	<u>Waterpipe cessation</u><br><br>Number of current waterpipe smokers before the intervention that were not current waterpipe smokers after the intervention<br><br><u>Waterpipe knowledge, attitudes, beliefs</u><br><br>“Is smoking shisha less harmful than smoking cigarettes?” Yes/No/I don’t know

Nakkash 2014<br><br>Funding: Qatar Research Fund for the National Priority Research Program NPRP 09-628-3-160<br><br>No conflicts of interest declared	Design: Cluster randomised controlled behavioural intervention<br><br>Year: 2011-2012	Qatar: 7th and 8th grade students<br><br>Lebanon: 6th and 7th grade students<br><br>Qatar: Intervention pre-test (N=146), post-test (N=127); control pre-test (N=107), post-test (N=105).<br><br>Lebanon: Intervention pre-test (N=1606), post-test (N=1271)<br><br>Setting: primary and secondary schools<br><br>Country: Lebanon and Qatar<br>Region/city: N/A (national intervention)	Ten sessions over ten weeks: four knowledge-focused, six skill-building (media literacy (1), decision making (2), refusal skills and social promise (3))	No intervention	Waterpipe cessation<br><br>Past-30 day waterpipe use<br><br>Waterpipe knowledge, attitudes and beliefs

Table 2: Characteristics of non-randomised quantitative studies

Study Name and Funding	Study Design	Participants, setting	Exposure	Control	Outcomes	Notes
Anjum 2008<br><br>Funding: World Health Organization, Eastern Mediterranean Regional Office (WHO-EMRO)	Design: Pre-post test<br><br>Year of study: 2006	Eligibility criteria: not mentioned<br><br>Characteristics:<br>- Pre-survey: N=646, 57% male, mean age 15 (SD 1), 49% class 9<br>- Post-survey: N=250, 37% male, mean age 15 (SD 1), 57% class 10<br>- Sig differences between gender and class ($p<0.001$), but not for age<br><br>Setting: educational institutions<br><br>Country: Pakistan<br>City/region: Karachi	Eight interactive WP health sessions, health hazards conveyed through handbills as take home messages	No control	No main outcome specified<br><br>Among all: current/ever WP prevalence<br>Among WP smokers: features of WP use, attitudes to cessation<br>Among non-WP smokers: intention to try WP<br>Among all: Health perceptions, social perceptions, influences, health hazards, associated diseases	Pre and post-test were different groups of Students<br><br>Post-test at two months follow up
Deshpande 2010<br><br>Funding: Nil	Design: Pre-post test<br><br>Year of study: 2008-2009	Eligibility criteria: commercial venues<br><br>Characteristics:<br>25 venues, of which two were waterpipe serving premises<br><br>Setting: waterpipe serving premises<br><br>Country: India<br>City/Region; Mumbai	Indian smokefree law	Five restaurants were used as a control	$PM_{2.5}$ measurements of indoor air quality before and after<br><br>Active smoker density before and after the ban using the number of people smoking and room volume	Post-test at approximately one year follow up
Essa-Hadad 2015<br><br>Funding: Center for Internet Research at the University of Haifa Israel	Design: Pre-post test<br><br>Year of study: 2007-2010	Eligibility criteria:<br>- Arab studying at a college or university in Israel<br>- Aged 18 years or older<br>- Access to internet, provides informed consent<br><br>Characteristics:<br>- Pre-test: N=356	Web-based program using the Questions Sharing and Interactive Assignments (QSIA) system with two parts: self-administered tobacco questionnaire, and tailored health education material via text and videos based on their responses	No control	<u>Waterpipe cessation</u><br><br>Past-7 day waterpipe use<br><br>Secondary: feasibility outcomes	Post-test at 1 month follow up

		- Post-test: N=225<br>- Mean age 25 (SD 5) years, 69% female, 47% Muslim, 73% religious, 73% undergraduate, 70% single<br><br>Setting: university<br><br>Country: Israel<br>City/region: N/A – across India				
Quadri 2014<br><br>Funding: not mentioned	Design: Pre-post test<br><br>Year of study: 2013	Eligibility criteria:<br>- non-science students<br>- aged 15-25 years<br><br>Characteristics:<br>N=1051, mean age 20 (SD 2), 57% male<br><br>Setting: six secondary school and six university colleges<br><br>Country: Saudi Arabia<br>City/region: Jazan	One lecture, distribution of educational brochure and a question-answer session	No control	Knowledge that waterpipe causes oral cancer<br><br>Acceptability, appeal and effectiveness of intervention	Post-test immediately after intervention
Salti 2015<br><br>Funding: International Development Research Centre, Canada. Grant number 105136-005	Design: Almost ideal demand system using cross-sectional data (Household Living Conditions Survey) containing price indices for different goods and services	Eligibility criteria: N/A<br><br>Characteristics:<br>N/A<br><br>Setting: N/A<br><br>Country: Lebanon<br>City/region: N/A – across Lebanon	Increased price of waterpipe tobacco	No control	Change in consumption of waterpipe (own price elasticity of demand)	

	Year of study: 2005					
Stamm-Balderjahn 2012<br><br>Funding: not mentioned	Design: Controlled pre-post test<br><br>Year of study: 2007-2008	Eligibility criteria:<br>- school students aged 12 to 19, attending a secondary general, intermediate, grammar, or comprehensive school<br>- apprentices aged 17 to 22 attending a part- or full-time vocational school<br><br>Characteristics:<br>- N=760<br>-Intervention group: n=382, 57% female, 35% secondary general and intermediate school<br>- Control group: n=378, 60% female, 38% secondary general and intermediate school,<br><br>Setting: 32 schools<br><br>Country: Germany<br>City/region: Berlin	- 2 hour long interactive presentation of the health consequences of smoking at a Berlin lung hospital.<br>- Starts with a 2-way conversation with a physician, informing the reasons for smoking, the mechanisms and risks of smoking with emphasis on health consequences and the role of advertising<br>- Followed by an interview with a patient suffering from a tobacco-related illness. Emphasis was on the consequences of the patients usually long-term smoking habit<br>- 2/3 students from each class had a lung function test and finger pulse oximetry<br>- A concluding group discussion about the test results (point above) and the students questions were answered	Control group who did not receive the interventi on, but details not describe d	Abstinence from waterpipe smoking initiation	Post-test at six months after intervention

Table 3: Characteristics of qualitative studies

**Study Name and Funding**	**Study Design**	**Participants, setting**	**Exposure**	**Control**	**Outcomes**	**Notes**
Highet 2011<br><br>Funding: Nil	Design: Pre-post test (qualitative)<br><br>Year of study: 2007-2008	Participants: Bangladeshi adult smokers (n=15) living in North and South of England who were smokers or had quit smoking in the previous 12 months	English smokefree law	No control	Substitution of waterpipe smoking	
Jawad 2013	Design: Post-legislation qualitative	Participants: 32 past-30 day, regular waterpipe smokers<br><br>Setting: London, UK	English smokefree law	No control	Waterpipe smoking behaviour	
Jawad 2014<br><br>Funding: City of Westminster Adult Services & Health Policy & Scrutiny Committee	Design: Post-legislation qualitative	Participants: 26 local government staff involved in enforcing against the waterpipe tobacco industry (trading standards=11, environmental health=7, public health=4, noise and licensing=2, planning enforcement=2).<br><br>Setting: London, UK	English smokefree law	No control	Waterpipe-serving premise compliance with English smokefree law	
Lock 2010<br><br>Funding: Islington Primary Care Trust, Goswell Road, London, N1, UK Other Funders: NHS.	Design: Pre-post test (qualitative) .	Setting: deprived North London neighbourhoods<br><br>Participants: 32 smokers selected from three ethnic groups, with approximately equal numbers of younger and older, male and female respondents<br><br>Participants: Pre-smokefree law, n=32, Ethnicity: 11 Somali, 13 Turkish, 8 White British/Irish; Age: 17 aged 18-30yrs, 9 aged 31-59yrs, 6 aged over 60yrs<br><br>Post, n=33 (23 were same as pre-smokefree	English smokefree law	No control	Change in smoking behavior, changes in the geographical location of smoking and its social impacts, and smoking illegally	Any other info

		law), Ethnicity: 13 Somali, 12 Turkish, 8 White British/Irish; Age: 18 aged 18-30yrs, 9 aged 31-59yrs, 6 aged over 60yrs				

Interventions for waterpipe tobacco smoking prevention and cessation: a systematic review

Mohammed Jawad, Sena Jawad, Reem K Waziry, Rami A Ballout, Elie A Akl

Supplementary Table S3: Risk of bias assessments using Cochrane (randomised studies) and GRADE (non-randomised studies) and CASP (qualitative studies) frameworks

Table 1: Risk of bias for randomised studies (Cochrane framework)

Study Name	Random sequence generation	Allocation concealment	Blinding or participants and personnel	Blinding of outcome assessment	Incomplete outcome data	Selective outcome reporting
Asfar 2014	Authors report using a computer random number generator.<br><br>Low risk of selection bias.	Insufficient information to permit judgement of 'Low risk' or 'High risk'..<br><br>Unclear risk of selection bias.	No blinding and outcome likely to be influenced by lack of blinding.<br><br>High risk of performance bias.	No blinding but outcome biochemically verified and unlikely to be affected by lack of blinding.<br><br>Low risk of detection bias.	Missing outcome data balanced across intervention groups, with similar reasons for missing data across groups.<br><br>Low risk of attrition bias.	The study protocol is available and all of the study's pre-specified (primary and secondary) outcomes that are of interest in the review have been reported in the pre-specified way<br><br>Low risk of reporting bias.
Dogar 2014	Authors report using a computer random number generator.<br><br>Low risk of selection bias.	Central allocation.<br><br>Low risk of selection bias.	No blinding and outcome likely to be influenced by lack of blinding.<br><br>High risk of performance bias.	No blinding but outcome biochemically verified and unlikely to be affected by lack of blinding.<br><br>Low risk of detection bias.	Missing outcome data balanced across intervention groups, with similar reasons for missing data across groups<br>Low risk of attrition bias.	Not all of the study's pre-specified primary outcomes have been reported (point abstinence at 4 weeks)<br>High risk of reporting bias.
Lipkus 2011	Authors report using a computer random number generator.<br><br>Low risk of selection bias.	Insufficient information to permit judgement of 'Low risk' or 'High risk'.<br><br>Unclear risk of selection bias.	Insufficient information to permit judgement of 'Low risk' or 'High risk'.<br>Unclear risk of performance bias.	Insufficient information to permit judgement of 'Low risk' or 'High risk'.<br><br>Unclear risk of detection bias.	Insufficient reporting of attrition/exclusions to permit judgement of 'Low risk' or 'High risk'<br><br>Unclear risk of attrition bias.	The study protocol is not available but it is clear that the published reports include all expected outcome.<br><br>Low risk of reporting bias.
Mohlman 2013	Insufficient information about the sequence generation process to permit judgement of 'Low risk' or 'High risk'.<br><br>Unclear risk of selection bias.	Insufficient information to permit judgement of 'Low risk' or 'High risk'.<br><br>Unclear risk of selection bias.	Insufficient information to permit judgement of 'Low risk' or 'High risk'.<br>Unclear risk of performance bias.	Insufficient information to permit judgement of 'Low risk' or 'High risk'.<br><br>Unclear risk of detection bias.	Missing outcome data balanced in numbers across intervention groups<br>Low risk of attrition bias.	One or more outcomes of interest in the review are reported incompletely so that they cannot be entered in a meta-analysis<br><br>High risk of reporting bias.
Nakkash 2014	Insufficient information about the sequence	Central allocation.	No blinding and outcome likely to be	No blinding and outcome is likely to be affected by	Insufficient reporting of attrition/exclusions to permit	Not all of the study's pre-specified primary outcomes have been reported

	generation process to permit judgement of 'Low risk' or 'High risk'.<br>Unclear risk of selection bias.	Low risk of selection bias.	influenced by lack of blinding.<br><br>High risk of performance bias.	lack of blinding.<br><br>High risk of detection bias.	judgement of 'Low risk' or 'High risk'<br><br>Unclear risk of attrition bias.	(point abstinence at 4 weeks)<br><br>High risk of reporting bias.

Table 2: Risk of bias non-randomised studies (GRADE framework)

**Study Name**	**Developing and applying appropriate eligibility criteria**	**Measurement of exposure**	**Measurement of outcome**	**Controlling for confounding**	**Completeness of data**
Anjum 2008	High risk<br><br>Non-random, convenience sampling of institutions (high risk).<br><br>Multistage sampling of participants, although no details given (unclear risk).	High risk<br><br>Authors do not report using a validated tool.	Unclear risk<br><br>Authors report using a pre-tested questionnaire but no adequate evidence of validation provided.	High risk<br><br>Authors do not report controlling for relevant confounders.	High risk<br><br>Authors provide no information about missing data, which appear apparent from the results tables.
Deshpande 2010	High risk<br><br>Mixture of random, convenience and purposive sampling.	Low risk<br><br>All venues are subject to the smokefree law and are hence 'exposed' to this health policy.	Low risk<br><br>Validated instrument for $PM_{2.5}$ measurement. Standardised protocol for taking measurements (i.e. in centre of venue, for 60 mins).	Unclear risk<br><br>Authors report controlling for confounders but without adequate details.	Low risk<br><br>Data are complete.
Essa-Hadad 2015	Low risk<br><br>Clear eligibility criteria.	Low risk<br><br>Authors report using a previously validated tool.	High risk<br><br>Authors do not report using a validated tool.	High risk<br><br>Authors do not report controlling for relevant confounders.	High risk<br><br>63.2% completion rate at follow up, missing responses excluded from analysis.
Quadri 2014	Low risk<br><br>Clear eligibility criteria. Participants selected through multi-stage cluster sampling.	High risk<br><br>Authors do not report using a validated tool.	High risk<br><br>Authors do not report using a validated tool.	High risk<br><br>Authors do not report controlling for relevant confounders.	Low risk<br><br>Authors report no drop outs; data are complete.
Salti 2015	Low risk	Low risk	Low risk	High risk	Low risk

	Nationally representative data.	Model based on the previously validated AIDS model.	Model based on the previously validated AIDS model.	Authors do not report controlling for relevant confounders.	Data are complete.
Stamm-Balderjahn 2012	High risk<br><br>Non-random, convenience sample with no eligibility criteria.	High risk<br><br>Authors do not report using a validated tool.	High risk<br><br>Authors do not report using a validated tool.	High risk<br><br>No controlling for confounding, but they assessed for interaction.	Low risk<br><br>Authors provide specific figures for missing data, suggesting low rates.

Table 3: Risk of bias for qualitative studies (CASP framework)

**Criteria**	**Highet 2011**	**Jawad 2013**	**Jawad 2014**	**Lock 2010**
Clear statement of aims	Yes<br><br>*"We use a 'life-world' lens to explore how male Bangladeshi smokers adapted to the English smoke-free legislation"*	Yes<br><br>*"The aims of the present study were to recruit university students in London to explore subjective opinions about waterpipe smoking in a large, ethnically diverse city, consolidating the existing epidemiological literature and adding to the foundation for evidence-based waterpipe tobacco control policies"*	Yes<br><br>"*This study aimed to gather knowledge and intelligence from London LA staff involved in legislation enforcement of the waterpipe industry and to gain insight into practical difficulties associated with applying existing legislation onto the waterpipe industry. By collating information from LA staff, this study aimed to gauge the scale of problems caused by the waterpipe industry and to recommend interventions to prevent uptake, to monitor the industry, and to enforce and extend legislation"*	Yes<br><br>*"To explore social and behavioural impacts of English smoke-free legislation (SFL) in different ethnic groups"*
Qualitative methodology appropriate	Yes	Yes	Yes	Yes
Research design appropriate	Yes<br><br>Design justified: *"Longitudinal qualitative research informs policy by providing detailed understandings of the contextual factors that may influence outcome among different groups over time…they seek to provide a deeper understanding of those factors which account for change over the course of*	No	No	No

	complex policy interventions"			
Recruitment strategy appropriate	Yes<br><br>Purposive recruitment, direct door-to-door and in public places<br><br>Bangladeshi sample in order to evaluate effect of smokefree law by ethnicity<br><br>No information on refusal to take part	Yes<br><br>Snowball sampling via key informants, social media, telephone and email<br><br>No information on refusal to take part	Yes<br><br>Purposive recruitment + snowball sampling, via email and telephone<br><br>Local authority staff sample in order to identify frontline legislative issues<br><br>Refusal to take part due to not finding suitable interview time	Yes<br><br>Purposive recruitment, using community contacts and direct public approach<br><br>Ethnically-diverse group in order to evaluate effect of smokefree law by ethnicity<br><br>Lost to follow up discussed (mainly Somali men), but were replaced in follow-up interview
Data collection appropriate	Can't tell<br><br>Setting: held in "*local venues*"<br>Methods: interviews and focus groups – unjustified, no information on topic guide, no information on whether methods were modified during study<br>Form of data unclear<br>Data saturation not reached as number of participants low	Yes<br><br>Setting: waterpipe cafes or university meeting rooms<br>Methods: focus group discussions – justified, no information on topic guide, no information on whether methods were modified during study<br>Form of data: audio, transcribed verbatim<br>Focus groups ceased when saturation reached	Can't tell<br><br>Setting: telephone discussions<br>Methods: interviews – unjustified, topic guide present, no information on whether methods were modified during study<br>Form of data: audio, transcribed verbatim<br>No discussion of data saturation	Can't tell<br><br>Setting: unclear<br>Methods: interviews – unjustified, topic guide present, no information on whether methods were modified during study<br>Form of data: audio, transcribed verbatim<br>No discussion of data saturation
Relationship between researcher and participants considered	Can't tell<br><br>Reflexivity mentioned but not considered in results or discussion: "*Throughout the study, the research team worked collaboratively to reach a consensus in interpretation and understanding, adopting an approach of critical self-reflection*"	No	No	Can't tell<br><br>Reflexivity mentioned but not considered in results or discussion: *"We used inductive, thematic analysis techniques,18 using a reflexive, iterative process of constant comparison involving team discussions…"*
Ethical issues considered	Yes<br><br>University of Edinburgh Research Ethics Committee	Yes<br><br>Imperial College Research Ethics Committee	No<br><br>No ethical approval sought	Yes<br><br>London School of Hygiene and Tropical Medicine research ethics

	Informed consent from participants	Informed consent from participants	Informed consent from participants	committee<br><br>Informed consent from participants
Data analysis sufficiently rigorous	Yes<br><br>In-depth description of analysis<br>Deductive and inductive theme derivation<br>Does not explain how data presented were selected from original sample<br>Sufficient data present to support findings<br>No information on dealing with contradictory data<br>Reflexivity present for analysis stage	Yes<br><br>In-depth description of analysis<br>Deductive theme derivation<br>Explains how data presented were selected from original sample<br>Sufficient data present to support findings<br>Contradictory data considered<br>No reflexivity present for analysis stage	No<br><br>No in-depth description of analysis<br>Deductive theme derivation<br>Does not explain how data presented were selected from original sample<br>Sufficient data present to support findings<br>No information on dealing with contradictory data<br>No reflexivity present for analysis stage	Can't tell<br><br>No in-depth description of analysis<br>Inductive theme derivation<br>Does not explain how data presented were selected from original sample<br>Sufficient data present to support findings<br>No information on dealing with contradictory data<br>Reflexivity present for analysis stage
Clear statement of findings	Yes<br><br>Explicit findings<br>Adequate discussion of both sides of argument<br>Adequate credibility (triangulation, more than one analyst)<br>Findings discussed in relation to original research question	Yes<br><br>Explicit findings<br>Adequate discussion of both sides of argument<br>Adequate credibility (triangulation, more than one analyst)<br>Findings discussed in relation to original research question	Can't tell<br><br>Explicit findings<br>Inadequate discussion of both sides of argument<br>Inadequate credibility (no triangulation, only one analyst)<br>Findings discussed in relation to original research question	Yes<br><br>Explicit findings<br>Adequate discussion of both sides of argument<br>Adequate credibility (triangulation, more than one analyst)<br>Findings discussed in relation to original research question
Value of research	Yes<br><br>Lack of information on waterpipe interventions	Yes<br><br>Lack of information on waterpipe interventions	Yes<br><br>Lack of information on waterpipe interventions	Yes<br><br>Lack of information on waterpipe interventions

Interventions for waterpipe tobacco smoking prevention and cessation: a systematic review

Mohammed Jawad, Sena Jawad, Reem K Waziry, Rami A Ballout, Elie A Akl

Supplementary Table S4: Individual results for each study

Table 1: Individual results for randomised studies

Study: Asfar 2014
Measurement: Self-reported verified by CO <10ppm
Follow-up time: 3 months

**Outcome**	**Intervention** Number with event	Number randomized	Number of participants with missing data	**Control** Number with event	Number randomized	Number of participants with missing data
Seven day point prevalence	11	27	6	7	23	4
Prolonged abstinence	12	27	6	7	23	4
Continuous abstinence	5	27	6	4	23	4

Study: Dogar 2014
Measurement: Verified by CO <10ppm
Follow-up time: 6 months

**Outcome**	**Intervention** Number with event	Number randomized	Number of participants with missing data	**Control** Number with event	Number randomized	Number of participants with missing data
Continuous abstinence in BSS+ group	10	27	7	13	70	6
Continuous abstinence in BSS group	53	118	2	13	70	6

Study: Lipkus 2011
Measurement: Self-reported questionnaire
Follow-up time: 6 months

**Outcome**	**Intervention** Number with event	Number randomized	Number of participants with missing data	**Control** Number with event	Number randomized	Number of participants with missing data
Not smoking WP in last 30 days among those who smoked monthly at baseline	18	37	9	11	42	9

Study: Mohlman 2013
Measurement: Self-reported survey
Follow-up time: One year

**Outcome**	**Intervention** Number with event	Number randomized	Number of participants with missing data	**Control** Number with event	Number randomized	Number of participants with missing data
Is smoking shisha less harmful than smoking cigarettes? (Yes/No/I don't know)	370	2789	Unclear	375	3141	Unclear
Number of current male waterpipe smokers before the intervention that were not current	14	250	Unclear	5	290	Unclear

waterpipe smokers after the intervention

Study: Nakkash 2014
Measurement: Self-reported survey
Follow-up time: Within a few days of intervention

**Outcome**	**Intervention**			**Control**		
	Number with outcome	Number randomized at baseline	Number of participants with missing outcome data	Number with outcome	Number randomized at baseline	Number of participants with missing outcome data
Past-30 day waterpipe use at post-test	122	270	Unclear	123	269	Unclear
Current waterpipe users at baseline who stopped post intervention	28	82	Unclear	21	92	Unclear
Increased waterpipe knowledge at post-test	336	413	Unclear	208	386	Unclear
"Healthy" waterpipe attitude at post-test	230	339	Unclear	172	327	Unclear

Table 2: Individual results for non-randomised quantitative studies

**Study Name**	**Outcome**	**Measurement**	**Point effect estimate with 95% Confidence Interval & P-value**	**Author interpretation**
Anjum 2008	• Assessed 2 months after exposure<br>• No main outcome specified<br><br>• Among all: current/ever WP prevalence<br>• Among WP smokers: features of WP use, attitudes to cessation<br>• Among non-WP smokers: intention to try WP<br>Among all: Health perceptions, social perceptions, influences, health hazards, associated diseases	Binary survey responses pre- and post-intervention	• Pre-test % vs post-test %<br><br>• Ever smoked WP (27 v 24) (p=0.37)<br>• Current WP smoking (17 v 14) (p=0.27)<br>• Share WP (76 v 68) (p=0.01)<br>• Use of other addictive substances (11 v 21), (p<0.001)<br>• Smokes usually at a café (75 v77) (p=0.56)<br>• Smoking with friends/family (91 v 85) (p=0.007)<br>• Smokes occasionally (76 v 71) (p=0.14)<br>• Want to quit WP (32 v 53) (p<0.001)<br>• Attempt to quit WP (28 v 27) (p=0.81)<br>• Try WP soon (non-smokers) (7 v 8) (p=0.69)<br>• Opinion whether WP is addictive (54 v 68) (p<0.001)<br>• WP is more addictive than cigarettes (11 v 32) (p<0.001)<br>• WP is more harmful than cigarettes (16 v 45) (p<0.001)	Perceptions regarding water pipe smoking changed significantly after intervention and the opinion regarding addiction associated with water pipe smoking improved. Highly significant difference was observed with regards to shisha being more addictive and harmful than cigarette smoking.<br><br>Social perceptions related to water pipe that it is more socially acceptable and part of our cultural heritage remain deep rooted and no significant difference was observed.<br><br>Majority of the students were of the opinion that shisha cafes play an important role in promoting shisha smoking. Most students said that shisha smoking is influenced by other people in close family circle smoking water pipe.

			• People smoking WP look cool (24 v 24) (p=0.99)<br>• People smoking WP have more friends (33 v 79) (p=0.78)<br>• Agree that WP is more socially acceptable compared to cigarettes (58 v 80) (p<0.001)<br>• Agree that WP smoking is our cultural heritage (29 v 58) (p<0.001)<br>• Agree that girls are more comfortable smoking WP than cigarettes (66 v 79) (p<0.001)<br><br>**Influences:**<br>• Immediate family/friends smoke WP (44 v 44) (p=0.99)<br>• Peer pressure (17 v 21) (p=0.19)<br>• Shisha café are playing an important role in promotion in WP (89 v 92) (p=0.18)<br>• WP smoking makes boys more attractive (19 v 20) (p=0.74)<br>• WP smoking makes girls more attractive (17 v 18) (p=0.73)<br>• **Health Hazards**<br>• Cardiovascular effects (24 v 10) (p<0.001)<br>• Respiratory effects (70 v 72) (p=0.55)<br>• Cancer (41 v 37) (p=0.25)<br>• Other bodily effects (18 v 23) (p=0.08)<br>• Oral infections (12 v 17) (p=0.06)<br>• None (9 v 6) (p=0.14)<br>• **Associated Diseases**<br>• Bladder cancer (19 v 33) (p<0.001)<br>• Bronchitis (50 v 56) (p=0.12)<br>• Oesophageal cancer (29 v 35) (p=0.07)<br>• Depression (25 v 23) (p=0.56)<br>• High blood pressure (31 v 16) (p<0.001)<br>• Parkinsons disease (5 v 6) (p=0.53)<br>• Ulcer (19 v 18) (p=0.72)<br>• Lip cancer (35 v 61) (p<0.001)<br>• Impaired pulmonary function (26 v 32) (p=0.07)<br>• Gum diseases (24 v 24) (p=0.99)<br>• Infections (35 v 34) (p=0.78)<br>• Infertility (10 v 38) (p<0.001)	Perceptions regarding health hazards associated with shisha smoking changed significantly after the health awareness sessions. The students attributed shisha smoking to all forms of cancers specifically those of lips, bladder and lung. Strong positive association was also observed with infertility, high blood pressure and cardiovascular problems.<br><br>Conclusion<br>The knowledge of the participating students regarding water pipe smoking improved to some extent after the health awareness sessions especially in terms of health hazards associated with water pipe. This study helped in changing their perceptions regarding health hazards associated with shisha smoking.
Deshpande 2010	• $PM_{2.5}$ measurements of indoor air quality before	SidePak AM510 Personal Aerosol	• PM2.5 decreased in all premises except hookah venues (mean 973 ug/m^3 pre ban to 1267 ug/m^3 post ban – 30%	This is possibly due to an exodus of smokers from their customary venues to hookah parlors, since these parlors were

	and after<br>• Active smoker density before and after the ban using the number of people smoking and room volume	Monitor	increase)<br>• Active smoker density decreased to zero in all premises except hookah venues, where it increased to 3.08 burning cigarettes per 100 cubic meters volume	clearly violating the law under the excuse that the flavored hookah's being served did not contain any nicotine.<br><br>Hookah parlors remained uniquely insulated from the ban's effect. It was apparent that cigarette smoking was not discouraged in the hookah enclosures.
Essa-Hadad 2015	• Primary: past-7 day waterpipe use<br>• Secondary: feasibility outcomes	Pre- and post-intervention survey	Past-7 day waterpipe use: 58.2% to 22.2% (p=0.01)<br><br>Satisfied or very satisfied with intervention: 97.8%<br>Recommend the intervention to a friend: 93.8%	The findings from the study suggest that a tailored Web intervention was found interesting and acceptable among Arab university students and seems promising in reducing nargila smoking.
Quadri 2014	Knowledge that waterpipe causes oral cancer	Pre- and post-intervention survey	Knowledge increased from 0.80 (SD 0.34) to 0.98 (SD 0.13).	The post intervention results showed a significant improvement in the knowledge of the respondents as the mean value obtained was fairly high.<br><br>The study effectively increased the knowledge and awareness among the youth about oral cancer per se and its prevention measures. Hence, giving a direction for further public health initiatives in this prone oral cancer region. Many educational programs should be conducted on a regular basis targeting a larger sector of the community.
Salti 2015	Change in waterpipe consumption	Price elasticity of demand	Price elasticity of demand: -1.45 (SD 0.007)<br><br>Price elasticity of demand of young adults (aged 15-30) -2.17	The expenditure data do not provide information on tobacco products consumed at other commercial establishments such as restaurants and cafes. Our measures of spending on shisha tobacco are therefore likely an underestimate the total spending on shisha tobacco by households.
Stamm-Balderjahn 2012	Abstinence from waterpipe smoking initiation	Pre- and post-intervention survey	Altogether, 23 students had taken up waterpipe smoking during the 6-month observation period: 5 in the intervention group, 18 in the control group. The difference was statistically significant (P<0,01). Compared to the control group, the nonsmokers (with respect to the waterpipe-only smokers) in the intervention group had a three and a half times likelihood of staying abstinent (OR: 3,64; SE: 0,52; 95% CI: 1,32 – 10,03).	N/A as this was additional information outside of the manuscript

Interventions for waterpipe tobacco smoking prevention and cessation: a systematic review

Mohammed Jawad, Sena Jawad, Reem K Waziry, Rami A Ballout, Elie A Akl

Table 3: Individual results for qualitative studies

Study Name	Outcome	Measurement	Selected quotes from participants	Author interpretation
Highet 2011	Substitution of waterpipe use	Qualitative interview	"I also use shisha as a substitute for coming off cigarettes, some people use nicotine patches and all that, I find shisha more effective . . . with shisha, a whole"<br><br>Watepripe use increased after the smokefree law for one participant – no waterpipe use for anyone else	These accounts suggest that smokers were using these products prior to, and after, the implementation of smoke-free legislation. Some regarded these practices as less harmful than smoking, while others framed them as an alternative way of weaning themselves off cigarettes:<br><br>However, some Bangladeshi smokers, old and young, appear to have increased their use of other forms of tobacco, such as shisha and paan, despite the former being included in smoke-free restrictions and the provision of specific guidance to this effect. Prior to the implementation of the legislation, there was widespread concurrent use of traditional cigarettes and indigenous tobacco products among Bangladeshi smokers. Since implementation, some smokers may be using such products as a substitute for smoking cigarettes and as an aid to smoking cessation, in the mistaken belief that these products are harmless. Thus, a modest reduction in cigarette consumption by some of our participants was counter-balanced by an increase in the use of other forms of tobacco.
Jawad 2013	Waterpipe smoking behavior after English smokefree law	Qualitative interviews post legislation	"Regarding the impact on waterpipe smoking of the 2007 smokefree law in England, opinions were divided into two broad categories: either there was no effect, or there was increased use as a result of the ban. Some participants adapted by smoking at home instead of at cafes, and subsequently increased their waterpipe consumption as it was more readily available and notably cheaper. Five years after the ban, participants described frequenting UK waterpipe cafes that flouted the smokefree law"	Of primary importance is the enforcement of waterpipe smoking legislation as directed by the World Health Organization Framework Convention on Tobacco Control. Other legislative issues that merit attention include appropriate taxation of waterpipe tobacco, enforcing the smokefree law to avoid carbon monoxide poisoning, and regulating the content of waterpipe tobacco
Jawad 2014	Waterpipe premise compliance with English smokefree law	Qualitative interviews post legislation	"Our enforcement policy basically says to give guidance, then send them a warning letter, then enforce. So they all know what they're doing is wrong and illegal, but they carry on doing it because a) they think they're going to get away with it or b) they'd rather take the fine and carry on with their business. I mean, I have one business which is right across the road, who says "What	Compliance with smoke-free law is generally poor, but unlike health warning labels or underage sales, is transiently compliant. Factors such as the cold weather, lack of regular monitoring from LA staff, peak times of trade, and low prosecution fines all encourage waterpipe premises to be noncompliant with smoke-free law. In one borough, fines ranged between £300 and £1,500. Many LA staff feel these

			do you want me to do? Without waterpipe I can't survive". Basically he has to break the law for his business to survive…that's what he says."<br><br>"I would say at the moment, I'm in court at least three times a month. Then if they plead 'not guilty' you have to prepare for the trial. You have to go back to court if sometimes they don't turn up…so it takes a lot of my time. It's difficult to put into hours or days, but at the moment I would say about 30% of what I'm doing is to do with waterpipe."<br><br>"A premises has forty people in there, if twenty of those are smoking and they paid fifteen pounds per waterpipe pipe – if they then get a fine of a hundred and fifty pounds, there's no deterrent for the premises because they can cover that in half a day.	fines are not designed for intentional and recurrent flouting of smoke-free law. Additionally, the prosecution process is labor and resource intensive.
Lock 2010	Change in smoking behavior, changes in the geographical location of smoking and its social impacts, and smoking illegally	Qualitative interviews pre- and post-smokefree law	"Some Somali respondents felt that smoking cessation services would not help as they focussed on cigarette use and did not address shisha smoking."<br><br>""For those who smoke Shisha they have to be home..... the gathering that used to take place in a restaurant takes place home a lot now." (Middle-aged Somali woman)"<br><br>"Somali women appeared to experience the greatest social impact of SFL. All Somali respondents discussed the traditional importance of shisha, with all but one of the Somali women currently or previously smoking shisha, while few smoked cigarettes. Despite an estimated 17% of Somali women in this community who admit to smoke, it is considered culturally unacceptable for Somali women to smoke, especially in public. Respondents said this was the custom rather than because of specific religious beliefs."<br><br>"Both Somali men and women agreed that the legislation has had a greater impact on women because of increased social restrictions. Before, SFL Somali women smokers could hire separate indoor smoking rooms in public shisha venues where they could socialise in private with friends. Women who continue to	It is important to understand the differences found between ethnic groups after SFL. Overall, the social impacts appeared most restrictive for young Somali women who, due to cultural sensitivity around female smoking, were often now unable to smoke in public where they might be seen and were thus taking measures to hide their smoking (including visiting illegal venues). Somali respondents also perceived that smoking cessation services were not culturally sensitive, focussing on cigarette, and this may have contributed to some of the ethnic differences seen in the lack of willingness to use cessation services.<br><br>The perceived stigma for some women associated with smoking outside in public since SFL may make already disadvantaged groups even more difficult to target or engage in future smoking cessation strategies

			smoke shisha say they feel that they now can only smoke in private homes or, if continuing to smoke publicly, by taking measures to conceal themselves, travelling away from their local community or smoking in illegal venues (box 5).<br><br>"For girls, they cannot sit outside. They feel a bit embarrassed. A friend - a family friend, like, someone might see them and tell the family. So what happened was, they put hoods on, a bit clothing on. Now they face on the wall, and they're just smoking. but they cannot sit there for a long time. And when they're, like, smoking the Shisha, they're not feeling comfortable.... I did it a couple of times, but it was at night time anyway. So I'm sure that my family aren't around. It was far away from where I live and I went out with a couple of friends, and even though it does matter, the way you dress up I just put my hood on like this, and.nobody's gonna see your face. ...I was not feeling comfortable. you know, before it was really okay, not anymore. It's the shame." (Young Somali woman)<br><br>""I think I told you, that these people will go underground. and, yes, they did. There were restaurants that had a lower floor and I think they will let only their regulars in...I sat there and I could easily say that the space occupied about 50 to 60 people and I wouldn't be able to see the person at the far corner." (Young Somali woman about shisha smoking)"<br><br>""..one place.it was a normal restaurant upstairs, which used to be a normal Shisha bar.and you'd have to go downstairs and there was a room in the basement, that was for Shisha smokers. And you can still find places that are the same as before, like, inside, but it's just the fact that you have to pay more for them just cause it's inside and that's not allowed." (Young Somali woman)"	
